# Supplementary material for: In Vitro Assessment of Antimicrobial Activity of Phytobiotics Composition towards of Avian Pathogenic Escherichia coli (APEC) and Other E. coli Strains Isolated from Broiler Chickens
Source: Antibiotics (Basel). 2022 Dec 15;11(12):1818. doi: 10.3390/antibiotics11121818 (PMC9774517; doi:10.3390/antibiotics11121818)
Supplement: Supplementary file 1 [file antibiotics-11-01818-s001.zip › antibiotics-2068603-SI.pdf]

In vitro assessment of antimicrobial activity of phytobiotics composition containing menthol, limonen, thymol, *p*-cymene, *trans*-anethole, methyl salicylate, terpinen-4-ol and γ-terpinene towards of Avian Pathogenic *Escherichia coli* (APEC) and other *E. coli* strains isolated from broiler chickens.

Karolina Chodkowska[1,2,*], Hubert Iwiński[2,3], Karolina Wódz[4], Tomasz Nowak[4], Henryk Różański

Table of content

Table S1. Antibiotic phenotype pattern, distribution of resistance genes, virulence genes and Multiple Antibiotic Resistance Index (MAR Index) amongst *Escherichia coli* isolates from poultry samples. 2

Table S2 Description of primer sets, annealing temperature and product size for antimicrobial genes . 4

Table S1. Antibiotic phenotype pattern, distribution of resistance genes, virulence genes and Multiple Antibiotic Resistance Index (MAR Index) amongst *Escherichia coli* isolates from poultry samples.

Sample origin	Antibiotic phenotype pattern	Resistance Genes	Virulence Genes	MAR index
chick APEC	AMX	*bla*$_{SHV}$	*astA, iss, irp2, cvi/cva, iucD, vat, iutA*	0.04
	AMX-CPH-DOX-OXY-FLR	*bla*$_{SHV}$, *tetA, tetB, floR*	*astA, iss, irp2, papC, iucD, vat. ompT*	0.2
	AMX-STR-NOR-DOX-OXY	*bla*$_{SHV}$, *aadA, strA/strB, tetA, tetB*	*astA, iss, irp2, papC, tsh, vat, ompT*	0.2
	AMX-CPH-STR-NOR-TR/SMX	*bla*$_{SHV}$ *,aadA, strA/strB*	*astA iss, irp2, papC, iucD, vat, iutA*	0.2
	AMX-STR-NOR-FLR-TR/SMX	*bla*$_{SHV}$, *aadA, strA/strB, floR*	*astA iss, irp2, cvi/cva, iucD, tsh, iutA*	0.24
	AMX-STR-DOX-OXY-LIN/SP-TR/SMX	*bla*$_{SHV}$, *aadA, strA/strB, tetA, tetB, sul1*	*astA iss, irp2, papC, cvi/cva, iucD, vat*	0.28
	AMX-CPH-GEN-NOR-DOX-OXY-TR/SMX	*bla*$_{SHV}$ *,aadB, tetA, tetB, sul1*	*astA iss, irp2, cvi/cva, iucD, tsh, iutA*	0.28
	AMX-CPH-STR-ENR-NOR-LIN/SP-TR/SMX	*bla*$_{SHV}$ *,aadA, strA/strB, sul1, sul2*	*astA, iss, irp2, cvi/cva, iucD, tsh, iutA*	0.28
	AMX-AMX/CL-STR-NOR-DOX-OXY-TR/SMX	*bla*$_{SHV}$, *aadA, strA/strB, tetA, tetB, sul1*	*astA, iss, irp2, papC, iucD, vat, iutA*	0.28
	AMX-GEN-STR-NOR-DOX-OXY-LIN/SP-TR/SMX	*bla*$_{SHV}$, *aadB, aadA, strA/strB, tetA, tetB, sul1, sul2*	*astA, iss, irp2, cvi/cva, iucD, tsh, iutA*	0.32
broiler chicken APEC	AMX	*bla*$_{SHV}$	*astA, iss, irp2, cvi/cva, iucD, vat, iutA*	0.04
	ENR-NOR	-	*astA, iss, irp2, cvi/cva, iucD, tsh, iutA*	0.08
	DOX-OXY	*bla*$_{SHV}$, *tetA, tetB*	*astA, iss, irp2, papC, cvi/cva, iucD, iutA*	0.08
	AMX-CPH-STR	*bla*$_{SHV}$ *,aadA, strA/strB*	*astA, iss, irp2, papC, cvi/cva, iucD, iutA*	0.12
	AMX-CPH-ENR-NOR	*bla*$_{SHV}$	*astA, iss, irp2, cvi/cva, iucD, tsh, iutA*	0.16
	AMX-CPH-STR-NOR-TR/SMX	*bla*$_{SHV}$, *aadA, strA/strB, sul1, sul2*	*astA, iss, irp2, papC, iucD, vat, iutA*	0.2
	AMX-CPH-DOX-OXY-FLR	*bla*$_{SHV}$, *tetA, tetB, floR*	*astA iss, irp2, papC, iucD, vat, ompT*	0.2
	AMX-CPH-STR-LIN/SP-TR/SMX	*bla*$_{SHV}$ *, aadA, strA/strB, sul1, sul2,dfrA1*	*astA, iss, irp2, cvi/cva, iucD, iutA, ompT*	0.2
	AMX-STR-NOR-FLR-TR/SMX	*bla*$_{SHV}$, *aadA, strA/strB, floR, sul1, dfrA1*	*astA, iss, irp2, cvi/cva, iucD, tsh, iutA*	0.2
	AMX-STR-NOR-DOX-OXY	*bla*$_{SHV}$, *aadA, strA/strB*	*astA, iss, irp2, papC, tsh, vat, ompT*	0.2
	NOR-DOX-OXY-FLR-LIN/SP	*tetA, tetB, floR*	*astA, iss, irp2, papC, cvi/cva, iucD, iutA*	0.2
	AMX-STR-DOX-OXY-LIN/SP-TR/SMX	*bla*$_{SHV}$, *aadA, strA/strB, tetA, tetB, sul1, sul2, sul3*	*astA, iss, irp2, papC, cvi/cva, iucD, vat*	0.24

	AMX-AMX/CL-STR-NOR-DOX-OXY-TR/SMX	*bla*$_{SHV}$, *aadA, strA/strB, tetA, tetB, sul1, sul2, dfrA10*	*astA, iss, irp2, papC, iucD, vat, iutA*	0.28
	AMX-CPH-GEN-NOR-DOX-OXY-TR/SMX	*bla*$_{SHV}$, *aadB, tetA, tetB, sul1, sul2, sul3*	*astA, iss, irp2, cvi/cva, iucD, tsh, iutA*	0.28
	AMX-CPH-STR-ENR-NOR-LIN/SP-TR/SMX	*bla*$_{SHV}$, *aadA, strA/strB, sul1, sul2, sul3, dfrA1*	*astA, iss, irp2, cvi/cva, iucD, tsh, iutA*	0.28
	AMX-CPH-STR-DOX-OXY-FLR-TR/SMX	*bla*$_{SHV}$, *aadA, strA/strB, tetA, tetB, floR, sul1, sul2, sul3*	*astA, iss, irp2, papC, iucD, tsh, iutA*	0.28
	AMX-CPH-STR-DOX-OXY-FLR-TR/SMX	*bla*$_{SHV}$, *aadA, strA/strB, tetA, tetB, floR, sul1, sul2*	*astA, iss, irp2, cvi/cva, iucD, tsh, vat*	0.28
	AMX-STR-DOX-OXY-FLR-LIN/SP-TR/SMX	*bla*$_{SHV}$, *aadA, strA/strB, tetA, tetB, floR, sul1, sul2, sul3, dfrA12*	*astA, iss, irp2, cvi/cva, iucD, tsh,iutA*	0.28
	AMX-CPH-STR-DOX-OXY-FLR-LIN/SP-TR/SMX	*bla*$_{SHV}$, *aadA, strA/strB, tetA, tetB, floR, sul2, sul3*	*astA, iss, irp2, papC, iucD, vat, iutA*	0.32
	AMX-GEN-STR-NOR-DOX-OXY-LIN/SP-TR/SMX	*bla*$_{SHV}$, *aadB, aadA, strA/strB, tetA, tetB, sul1, sul2, sul3*	*astA, iss, irp2, cvi/cva, iucD, tsh, iutA*	0.32
	AMX-STR-ENR-NOR-DOX-OXY-FLR-LIN/SP	*bla*$_{SHV}$, *aadA, strA/strB, tetA, tetB, floR*	*astA, iss, irp2, papC, iucD, tsh, vat, iuaT*	0.32
	AMX-CPH-STR-ENR-NOR-DOX-OXY-FLR-TR/SMX	*bla*$_{SHV}$, *aadA, strA/strB tetA, tetB , floR sul1, sul2, sul3*	*astA, iss, irp2, papC, iucD, vat, iutA*	0.36
	AMX-CPH-STR-ENR-NOR-DOX-OXY-FLR-TR/SMX	*bla*$_{SHV}$, *aadA, strA/strB, tetA, tetB, floR sul1, sul2, sul3*	*astA, iss, irp2, papC, iucD, tsh, vat,*	0.36
	AMX-CPH-ENR-NOR-DOX-OXY-FLR-LIN/SP-TR/SMX	*bla*$_{SHV}$, *tetA, tetB, floR dfrA1, dfrA10*	*astA, iss, irp2, cvi/cva, iucD, tsh, iutA*	0.36

Letter abbreviations correspond to the individual antibiotics according to list: amoxicillin (AMX), amoxicillin and clavulanic acid (AMX/CL), , cefapirin (CPH), gentamicin (GEN), nstreptomycin (STR), enrofloxacin (ENR), norfloxacin (NOR), docycycline (DOX), oxytetracycline (OXY), florfenicol (FLR), lincomycin/specinicin (LIN/SP), trimethoprim-sulfamethoxazole (TR/SMX).

Table S2 Description of primer sets, annealing temperature and product size for antimicrobial genes

**Multiplex PCR or single PCR**	**Gene/antibiotic**	**Primer sequences 5' - 3'**	**Annealing temperature**	**PCR Product Size (bp)**
**Multiplex** 1	*aadA*<br>streptomycin	F - GTG GAT GGC GGC CTG AAG CC<br>R -AAT GCC CAG TCG GCA GCG	63 °C	525 bp
**Multiplex** 1	*strA*/*strB*<br>streptomycin	F - ATG GTG GAC CCT AAA ACT CT<br>R - CGT CTA GGA TCG AGA CAA AG	63 °C	893 bp
**Multiplex 2**	*aphA1*<br>neomycin	F - ATG GGC TCG CGA TAA TGT C<br>R - CTC ACC GAG GCA GTT CCA T	55 °C	634 bp
**Multiplex 2**	*aphA2*<br>neomycin	F - GAT TGA ACA AGA TGG ATT GC<br>R - CCA TGA TGG ATA CTT TCT CG	55 °C	347 bp
**Multiplex 2**	*aadB*<br>gentamicin	F - GAG GAG TTG GAC TATGGA TT<br>R - CTT CAT CGG CAT AGT AAA AG	55 °C	208 bp
**Multiplex 3**	*tetA*<br>tetracycline	F - GGC GGT CTT CTT CAT CAT GC<br>R -CGG CAG GCA GAG CAA GTA GA	63 °C	502 bp
**Multiplex 3**	*tetB*<br>tetracycline	F - CGC CCA GTG CTG TTG TTG TC<br>R -CGC GTT GAG AAG CTG AGG TG	63 °C	173 bp
**Multiplex 4**	*sul1*<br>sulfamethoxazole	F - CGG CGT GGG CTA CCT GAA CG<br>R - GCC GAT CGC GTG AAG TTC CG	66 °C	433 bp
**Multiplex 4**	*sul2*<br>sulfamethoxazole	F - CGG CAT CGT CAA CAT AAC CT<br>R -TGT GCG GAT GAA GTC AGC TC	66 °C	721 bp
**Single PCR**	*sul3*<br>sulfamethoxazole	F - GGGAGCCGCTTCCAGTAAT<br>R- TCCGTGACACTGCAATCATTA	60 °C	500 bp
**Single PCR**	*dfrA1*<br>*trimethoprim*	F - CAATGGCTGTTGGTTGGAC<br>R - CCGGCTCGATGTCTATTGT	62 °C	253 bp
**Single PCR**	*dfrA10*<br>*trimethoprim*	F - TCAAGGCAAATTACCTTGGC<br>R - ATCTATTGGATCACCTACCC	59°C	433 bp
**Single PCR**	*dfrA12*<br>trimethoprim	F - TTCGCAGACTCACTGAGGG<br>R - CGGTTGAGACAAGCTCGAAT	63 °C	330 bp
**Single PCR**	*floR*<br>*florfenicol*	F - CACGTTGAGCCTCTATATGG<br>R - ATGCAGAAGTAGAACGCGAC	61 °C	888 bp
**Multiplex 5**	bla_{TEM}<br>ampicillin	F - TTAACTGGCGAACTACTTAC<br>R - GTCTATTTCGTTCATCCATA	55 °C	247 bp
**Multiplex 5**	bla_{SHV}<br>amoxicillin	F - AGGATTGACTGCCTTTTTG<br>R - ATTTGCTGATTTCGCTCG	55 °C	393 bp
**Multiplex 5**	$bla_{CMY\text{-}2}$<br>ceftiofur	F - GACAGCCTCTTTCTCCACA<br>R - TGGACACGAAGGCTACGTA	55 °C	1000 bp
**Single PCR**	$bla_{PSE\text{-}1}$<br>ampicillin	F- GCAAGTAGGGCAGGCAATCA<br>R- GAGCTAGATAGATGCTCACAA	60 °C	461 bp
**Single PCR**	$bla_{CTX\text{-}M}$	F- CGCTTTGCGATGTGCAG<br>R- ACCGCGATATCGTTGGT	60 °C	585 bp
